# Anodic Titanium Dioxide Nanotubes for Magnetically Guided Therapeutic Delivery

**DOI:** 10.1038/s41598-019-49513-2

**Published:** 2019-09-17

**Authors:** Morteza Hasanzadeh Kafshgari, Delf Kah, Anca Mazare, Nhat Truong Nguyen, Monica Distaso, Wolfgang Peukert, Wolfgang H. Goldmann, Patrik Schmuki, Ben Fabry

**Affiliations:** 1 Department of Physics, Biophysics Group, University of Erlangen-Nuremberg, 91052 Erlangen, Germany; 2 Department of Materials Science and Engineering, WW4-LKO, University of Erlangen-Nuremberg, Martensstrasse 7, 91058 Erlangen, Germany; 3 Institute of Particle Technology, University of Erlangen-Nuremberg, 91058 Erlangen, Germany; 4 Present Address: Department of Engineering Physics, Polytechnique Montreál, Montreál, Quebec H3C3A7 Canada

**Keywords:** Synthesis and processing, Drug delivery, Magnetic properties and materials

## Abstract

Hollow titanium dioxide (TiO_2_) nanotubes offer substantially higher drug loading capacity and slower drug release kinetics compared to solid drug nanocarriers of comparable size. In this report, we load TiO_2_ nanotubes with iron oxide nanoparticles to facilitate site-specific magnetic guidance and drug delivery. We generate magnetic TiO_2_ nanotubes (TiO_2_NTs) by incorporating a ferrofluid containing Ø ≈ 10 nm iron oxide nanoparticles in planar sheets of weakly connected TiO_2_ nanotubes. After thermal annealing, the magnetic tubular arrays are loaded with therapeutic drugs and then sonicated to separate the nanotubes. We demonstrate that magnetic TiO_2_NTs are non-toxic for HeLa cells at therapeutic concentrations (≤200 µg/mL). Adhesion and endocytosis of magnetic nanotubes to a layer of HeLa cells are increased in the presence of a magnetic gradient field. As a proof-of-concept, we load the nanotubes with the topoisomerase inhibitor camptothecin and achieve a 90% killing efficiency. We also load the nanotubes with oligonucleotides for cell transfection and achieve 100% cellular uptake efficiency. Our results demonstrate the potential of magnetic TiO_2_NTs for a wide range of biomedical applications, including site-specific delivery of therapeutic drugs.

## Introduction

Due to their small size and high specific surface area, drug-functionalized nanoparticles – so-called nanocarriers – can be effective means to deliver therapeutic agents to the site of action in the body^1,2^. Nanocarriers can be modified by different functional moieties to target specific cancer cells and subcellular compartments for releasing the drug cargo only at the destination site^3,4^. Moreover, by tuning the size and shape of the nanocarriers, it is possible to increase their accumulation in the microenvironment, e.g. of malignant solid tumors^5,6^.

Titanium dioxide nanotubes (TiO_2_NTs) are a new class of biocompatible elongated nanocarriers^7,8^. Recently, the fabrication of well-separated, single, uniform TiO_2_NTs by sonication of anodic nanotube arrays has been reported^9^. Individual TiO_2_NTs are highly versatile drug delivery systems due to their large loading capacity and straight-forward functionalization, e.g. with polymers or linkers that respond to pH or light^10–12^. The specific hollow morphology of TiO_2_NTs (closed at one end) provides improved control over therapeutic loading and release compared to solid nanocarriers. Moreover, due to their elongated shape with a high aspect ratio, TiO_2_NTs are subjected to hemodynamic forces and torques in the blood stream that facilitates their drift towards endothelial walls, which improves margination in the microenvironment of solid tumors^13,14^.

In this report, we describe how TiO_2_NTs can be further functionalized with iron oxide nanoparticles for magnetically guiding and accumulating the nanocarriers to the desired site of action, similar to solid iron oxide nanoparticles.

## Results and Discussion

**Figure 1:**
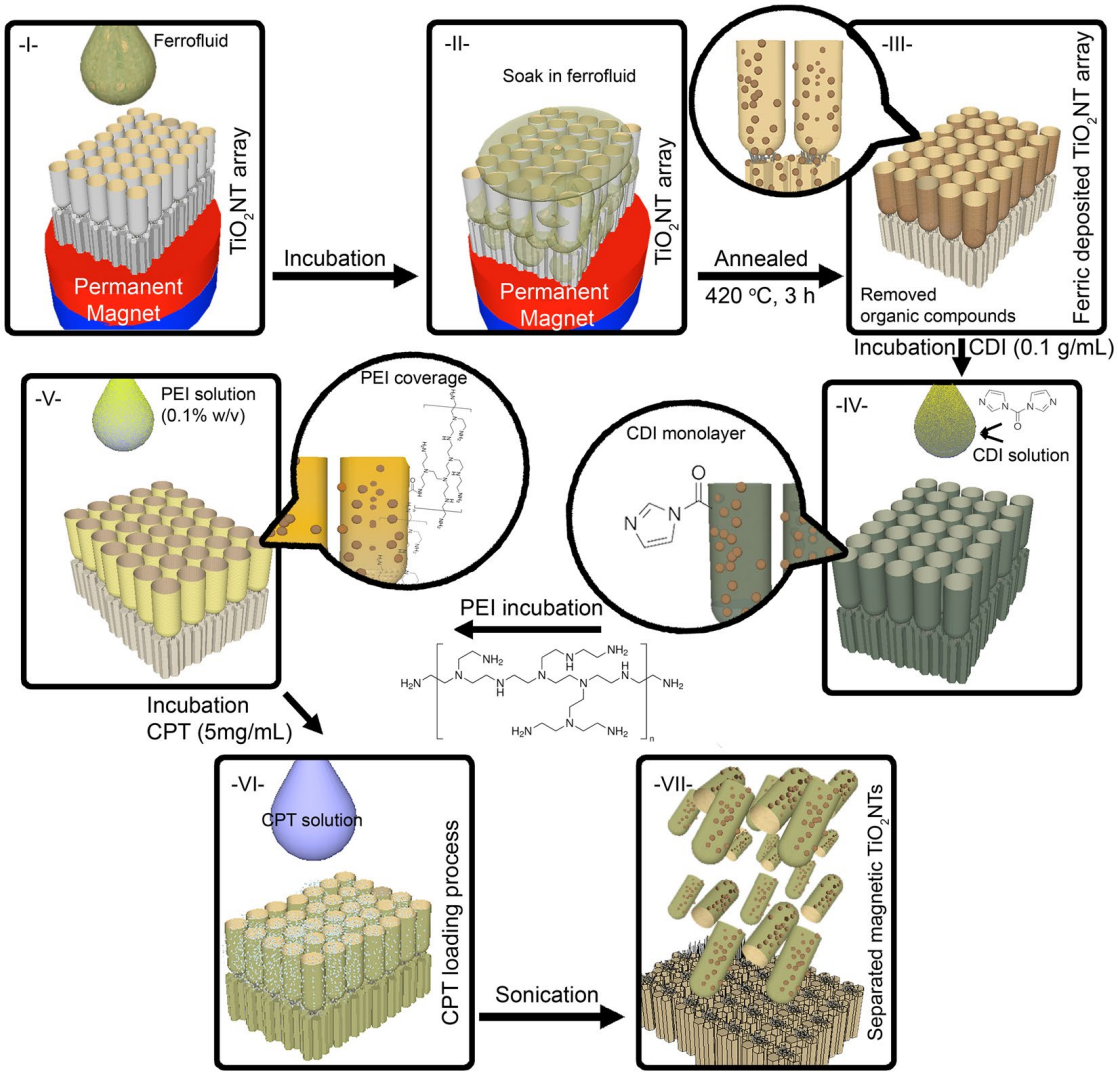
Schematics of the fabrication of camptothecin-loaded magnetic TiO_2_NTs; (I, II) The incubation of ferrofluid with a triple-layer anodic TiO_2_NT array under the gradient magnetic field, (III) thermal treatment of the ferrofluid deposited TiO_2_NT array to remove stabilizers and other chemicals of the ferrofluid, (IV) generation of carbonyldiimidazole (CDI) monolayer, (V) conjugation of polyethylenimine (PEI) on the surface of nanotubes, (VI) incubation of anticancer drug camptothecin (CPT) with ferric/TiO_2_NT arrays, and (VII) sonication of the array to prepare camptothecin-loaded magnetic TiO_2_NTs. Note that, other therapeutics (i.e., oligonucleotides) can be also incubated with the array at this loading step.

**Figure 2:**
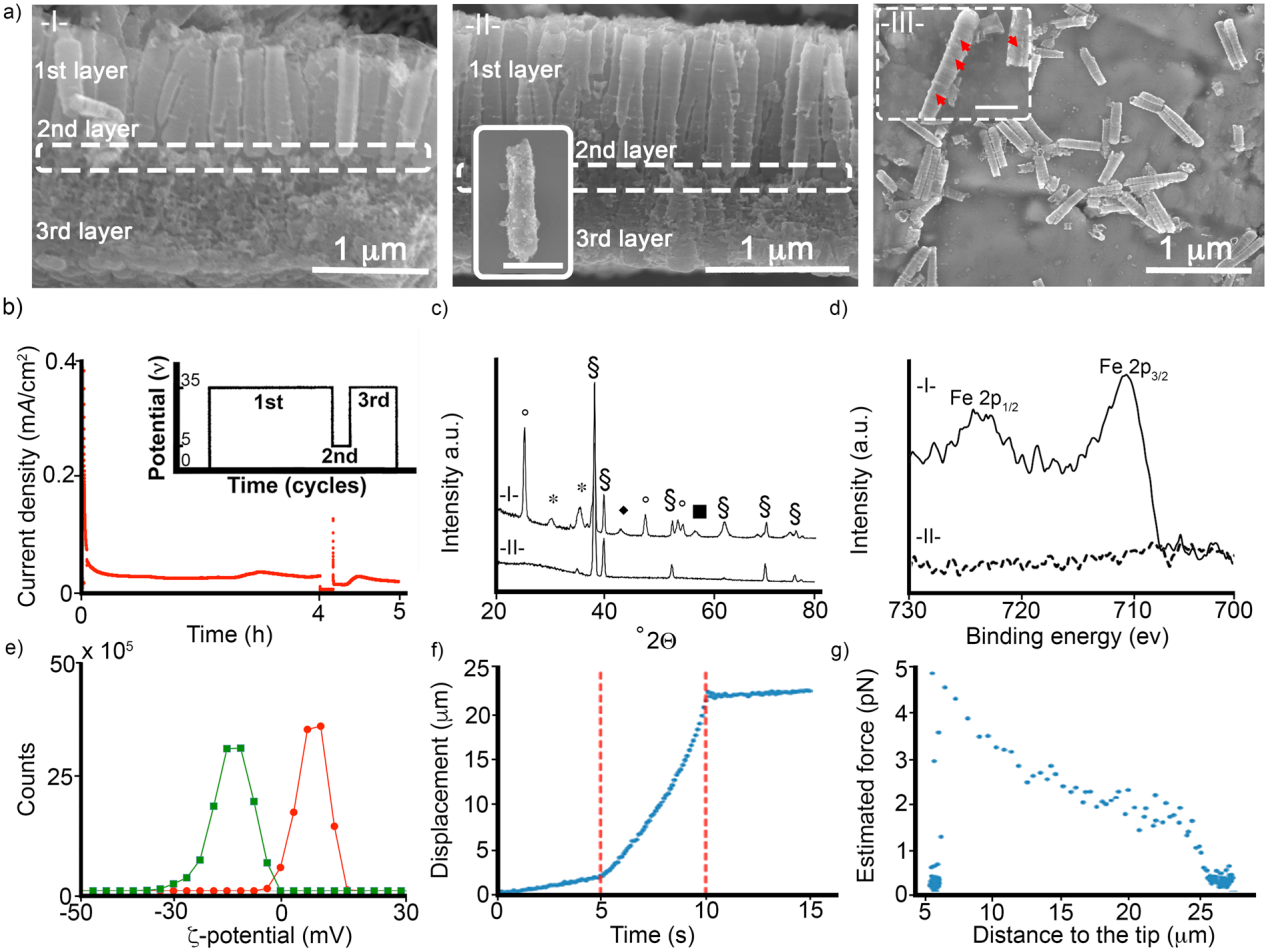
(**a**) Representative SEM micrographs of (I) anodic TiO_2_NT arrays, (II) ferric/TiO_2_NT array and (III) magnetic TiO_2_NTs. The inset micrographs show the presence of ferric nanoparticles (indicated by red arrows) on the surface of magnetic TiO_2_NTs. Scale bars of images are 500 nm. Figure S1 shows representative top view SEM micrographs of (I) anodic TiO_2_NT arrays and (II) ferric/TiO_2_NT array. (**b**) Current density profile of the electrochemical anodization of TiO_2_NT arrays. Inset graph shows the potential profile (an ultrafast switching voltage). (**c**) XRD spectra of ferric/TiO_2_NTs (I) and TiO_2_NTs (II); the signs represent (°) anatase, (*) magnetite, (§) titanium, (■) hematite, and (♦) iron oxide. (**d**) XPS spectra of the magnetic TiO_2_NTs (I) and TiO_2_NTs (II). (**e**) ζ-Potential (mV) of TiO_2_NTs (green line) and magnetic TiO_2_NTs (red line). (**f**) The measured displacement of magnetic TiO_2_NTs under magnetic force generated by the magnetic tweezer device. Red dashed lines (at 5 and 10 sec) show the period that the magnetic tweezer device was active. (**g**) The estimated force (pN) applied to magnetic TiO_2_NTs based on their distance to the tip.

Nanofabrication and post-fabrication of polyethylenimine-coated ferric anodic TiO_2_NTs (magnetic TiO_2_NTs) loaded with camptothecin is summarized in Fig. 1. To obtain TiO_2_NTs, a time-varying electrochemical anodization in the presence of ammonium fluoride (0.27M) enriched glycerol/water (60/40 v/v) electrolyte was employed to fabricate multilayer anodic TiO_2_NT arrays (Fig. 2a-I). The anodization protocol to form the multilayer stacks was adjusted by applying three voltage steps (35V for 240 min, 5V for 10 min, and 35 V for 60 min) to generate a fragile layer with defined breaking points (Fig. 2b)^15^. The formation of such multilayer stacks was controlled by alterations of the electric field, which determines the growth mechanism and hence the final structure and morphology of TiO_2_ tubular arrays^9,16^.

We then deposited ferric nanoparticles by filling a ferrofluid (Ferrofluid-EFHI, ferric nanoparticles Ø ≈ 10 nm) into the fabricated tubular array (average pore size 187.81 ± 4.2 nm; Fig. S1), using a permanent NdFeB disc magnet (Ø: 2 cm, length: 1 cm, 1.42T) placed directly under the array. Afterwards, organic stabilizers of the ferric solution were removed from the tubular array by thermal annealing (at 420 °C, for 3 h in air). The resulting morphology of the tubular arrays was investigated by SEM imaging (Fig. 2a-II). X-ray powder diffraction (XRD) measurements were then carried out to analyze the crystalline structure of TiO_2_NTs and the ferric nanoparticles (Fig. 2c). The thermal annealing changed the amorphous nature of the arrays to anatase. However, the crystalline structure of the ferric nanoparticles remained unaltered after the annealing process^16^.

X-ray photoelectron spectroscopy (XPS) characterization was employed to confirm the presence of iron in the tubular TiO_2_ nanostructures (Fig. 2d, peaks at 711 eV (Fe2p_3/2_) and 723 eV (Fe2p_1/2_))^17^. The high-resolution spectra indicate the presence of iron as Fe^+^3, which is in accordance with XRD measurements. As illustrated in Figs S2 and S3, the XPS depth profiles (up to 100 nm) show the deposition of ferric nanoparticles in the tubular array.

Magnetic TiO_2_NT arrays were functionalized prior to separation by sonication with a monolayer of carbonyldiimidazole, which served as a linker to bind polyethylenimine (Figs 1 and 2). Polyethylenimine trapped the nanoparticles inside the nanotubes and minimized their loss during the subsequent sonication process (~90 min) that separated the magnetic TiO_2_NTs from the array. As shown in Fig. 2a-III, the average length of the nanotubes after sonication is around 1236 ± 35 nm. The surface charge of the functionalized nanocarriers is +8.97 mV compared to −14 mV for non-magnetic TiO_2_NTs without polyethylenimine functionalization at pH 6.6 (Fig. 2e).

A previous study showed that up to 75% of the separated particles after sonication contained individual TiO_2_NTs, while the remainder contained double and triple connected tubes^9^. In the case of magnetic nanotubes, the majority (≥85%) of the particles contained double and triple connected tubes (Fig. 2a-III). We attribute the incomplete separation to the polyethylenimines coating and long annealing procedure, which generated stronger intertubular connections such as ridges (Fig. S4). Therefore, it is also crucial to adjust the power and time of the sonication process to avoid loss of iron nanoparticles on the one hand, and to achieve a sufficient breakup and separation of the nanocarrier array on the other hand.

The magnetic properties of the TiO_2_NTs were evaluated using a magnetic tweezer, whereby the nanocarriers were dispersed in viscous silicone oil (viscosity 0.3 Pa.s), and the speed of their movement in response to a magnetic gradient field was measured (Fig. 2f)^18^. The generated force was computed using Stokes’ law generalized for cylindrical objects (Fig. 2g)^19^. As the number of nanotubes in a given particle was not discernible by bright field microscopy, we assumed that each particle contained an aggregate of two nanotubes, corresponding approximately to a cylinder of 250 nm in radius and 1200 nm in height. Further, we assumed that the cylinders align in the direction of their movement to minimize viscous drag. At a distance of 20 µm from the tweezer tip, we estimated a force of 2 pN. This force was lower than that expected for commercially available magnetic beads (i.e., Invitrogen Dynabeads M-450), which would amount to 54 pN if the bead volume was scaled down accordingly, assuming a linear dependence of force with volume^20^. Increasing the loading of the nanotubes with magnetic nanoparticles was expected to increase the achievable force, but this needed to be balanced by the necessarily diminished loading of the nanotubes with therapeutic agents.

**Figure 3:**
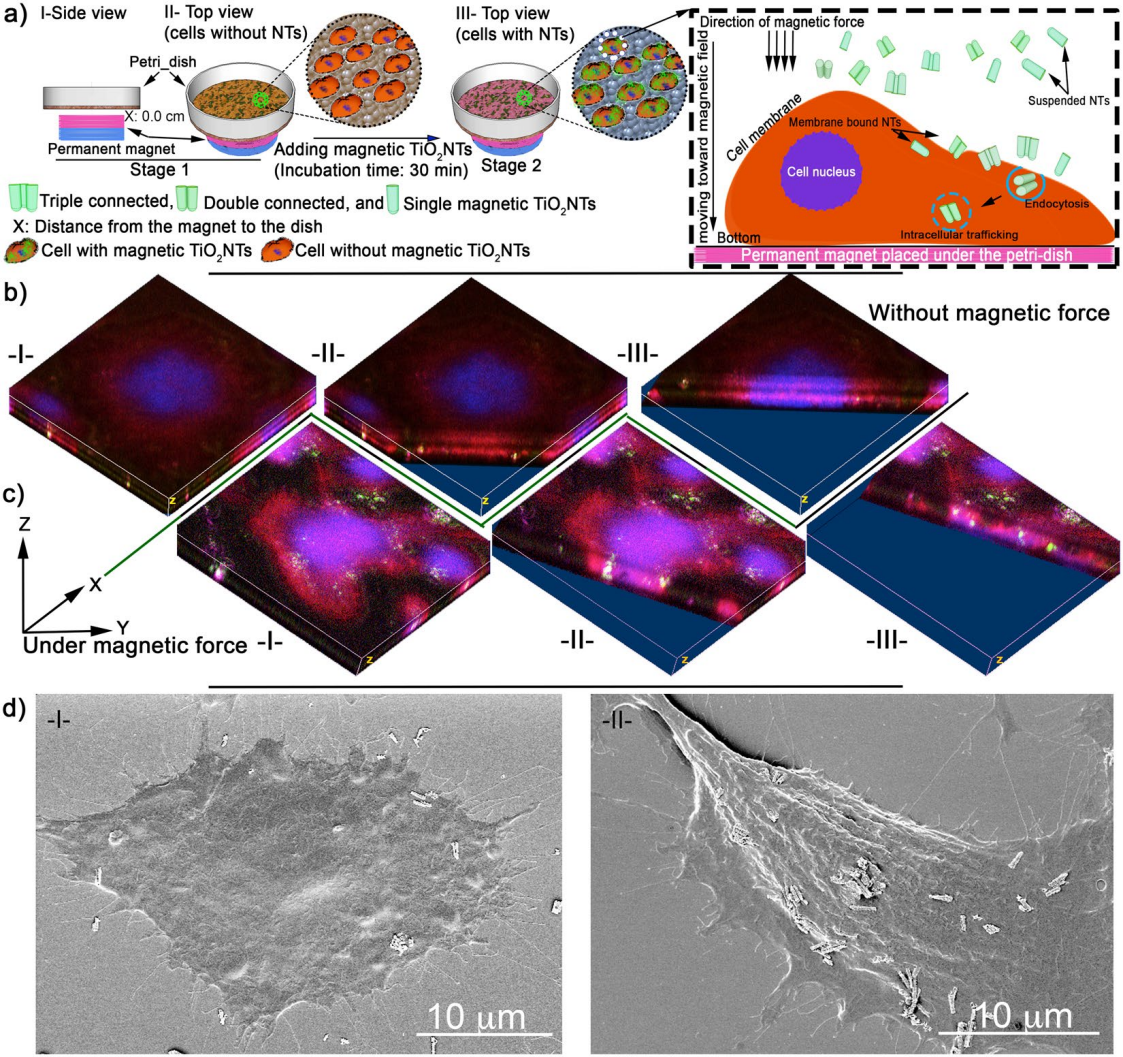
(**a**) Schematic of enhanced cellular uptake of magnetic TiO_2_NTs under the influence of a gradient magnetic field. The cells were incubated with FITC-conjugated magnetic TiO_2_NTs for 30 min and then washed thoroughly to remove unbound nanocarriers. The overall distance between the permanent magnet and the cell surface was ≈2.7 mm. Representative micrographs of FITC-conjugated magnetic TiO_2_NTs without (**b**) and with (**c**) application of a gradient magnetic field imaged by progressive Z-stack confocal microscopy (ImageJ processed 3D volume micrographs). Roman numbers, indicate images at different XY distances, illustrate internalized nanocarirers (green). Cell nuclei and actin were stained with DRAQ5^TM^ (shown in blue) and phalloidin-TRITC (red) respectively, and FITC-conjugated magnetic TiO_2_NTs are shown in green. (**d**) Representative SEM micrographs of internalizing magnetic TiO_2_NTs without (I) and with (II) magnetic force.

To study the effects of an external magnetic force on cellular uptake, fluorescein isothiocyanate (FITC) was conjugated on the surface of magnetic TiO_2_NTs. Under the effect of static magnetic forces generated by a permanent NdFeB disc magnet (Ø: 2 cm, length: 1 cm, 1.42T) positioned beneath the cell culture dish, FITC-conjugated magnetic TiO_2_NTs (0.2 mg/mL) incubated with HeLa cells (a cervical cancer cell line) settled rapidly on the cell surface (~6 NTs per cell on average) and were subsequently internalized in subcellular compartments as evaluated by laser-scanning confocal microscope and shown by SEM micrographs (Figs 3, S5 and S6). In contrast, in the absence of magnetic force, the average number of cell-bound nanocarriers was reduced to ~2 NTs per cell. Similar results were also achieved with oligonucleotide-loaded magnetic TiO_2_NTs, where we found a 100% cellular binding after 30 min under the influence of magnetic force compared to approximately 10% in the absence (Fig. S7).

**Figure 4:**
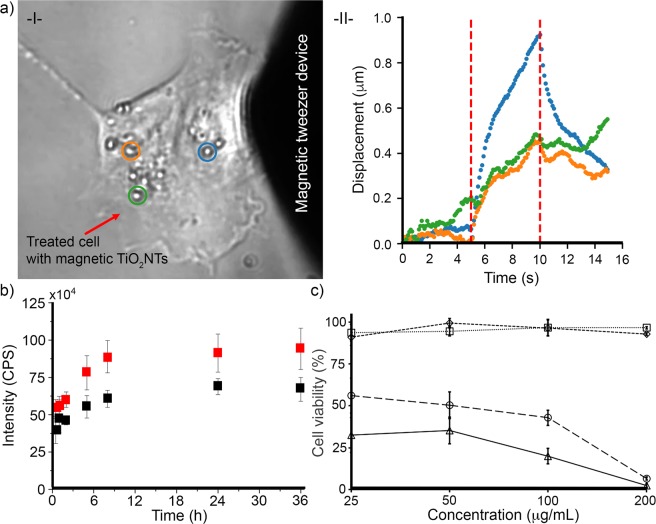
(**a**) Displacement of the magnetic TiO_2_NTs internalized in a single HeLa cell by employing magnetic force, -I- a representative light microscopic image of a treated cell and -II- measured displacement of representative magnetic TiO_2_NTs (indicated by blue, orange, and green cycles). Red dashed lines (at 5 and 10 sec) show the period that the magnetic tweezer device was active. (**b**) Cumulative camptothecin release profiles from camptothecin-loaded magnetic TiO_2_NTs (black squares) and camptothecin-loaded TiO_2_NTs (red squares). Release medium: PBS, pH 7.4, T = 37 °C. (**c**) Effects of anticancer camptothecin delivery, using magnetic TiO_2_NTs under the influence of a gradient magnetic field. HeLa cells incubated for 30 min with nanocarriers under the influence of a static gradient magnetic field were thoroughly washed in order to eliminate the unbound nanocarriers and then incubated with fresh cell culture medium (without nanocarriers) for 72 h. Cell viability of HeLa cells treated with camptothecin-loaded magnetic TiO_2_NTs (Δ) and empty magnetic TiO_2_NTs (□) under the influence of a gradient magnetic field, as well as camptothecin-loaded magnetic TiO_2_NTs (◯) and empty magnetic TiO_2_NTs (◊) without employing the gradient magnetic field were measured after 72 h of incubation (T = 37 °C, 5% CO_2_).

When we applied magnetic force to membrane-bound nanotubes with a magnetic tweezer (Fig. 4a and video in Section S5)^20^, we measured displacements of up to one micrometer, demonstrating that the nanotubes kept their magnetic properties (Fig. S8) after attachment to the cells.

To test if magnetic nanotubes can be further loaded with therapeutic drugs, the magnetic tubular arrays were incubated overnight with 5 mg/mL of camptothecin, a topoisomerase inhibitor used for cancer treatment, and then sonicated as described above. After washing the drug-loaded nanocarriers twice to remove weakly bound drugs, a fluorometer was used to determine the amount of loaded camptothecin, yielding 244.31 μg/mg of magnetic nanotubes, compared to 348.97 µg/mg for non-magnetic nanotubes. We next measured the release of camptothecin from magnetic TiO_2_NTs and non-magnetic TiO_2_NTs in PBS over 36 h at 37 °C (pH 7.4). The release kinetics showed a fast release of 60% of the bound camptothecin during the first 30 min, followed by a slow release of the remaining drug over 36 h. The release kinetics was similar for both magnetic and non-magnetic nanotubes (Fig. 4b).

To evaluate the applicability of camptothecin-loaded TiO_2_NTs as a drug delivery system, we employed an extra washing step to remove the weakly bound camptothecin. Cytotoxicity from camptothecin-loaded and unloaded magnetic TiO_2_NTs with and without static magnetic force was measured using a 3-(4,5-dimethyl-2-thiazolyl)-2,5-diphenyltetrazolium bromide (MTT) assay (Fig. 4c). HeLa cells were incubated at different concentrations of camptothecin-loaded and unloaded (empty) magnetic TiO_2_NTs for 30 min under static magnetic force, followed by the exchange of culture medium to remove unbound nanocarriers. After 72 h of incubation, the cell viability of HeLa cells incubated at low concentration of camptothecin-loaded magnetic TiO_2_NTs under the influence of a gradient magnetic field was significantly lower compared to that of the treatment without magnetic force. The presence or absence of a gradient magnetic field did not alter the viability of cells incubated with empty magnetic TiO_2_NTs (at the used concentrations), regardless of concentration (Fig. 4c), indicating that magnetic TiO_2_NTs were biocompatible at the used concentration for at least 72 h of incubation.

## Conclusions

In summary, we describe the fabrication of TiO_2_ nanocarriers designed for magnetic targeting and therapeutic delivery. We show that the movement of nanocarriers can be controlled in the presence of gradient magnetic fields, and that an increased internalization of magnetic TiO_2_ nanocarriers into HeLa cells can be achieved in the presence of a static gradient magnetic field. Treated HeLa cells remain viable after incubation for 72 h with empty magnetic TiO_2_ nanocarriers (≤200 µg/mL). As a proof of concept for targeted therapeutic drug treatment, we demonstrate site-specific magnetically enhanced delivery of camptothecin and oligonucleotides.

## Materials and Methods

### Materials

Titanium foils (99.6% purity, thickness 0.125 mm) were purchased from Advent Research Materials Ltd. (Oxford, UK). Dulbecco’s modified Eagle’s medium (DMEM), phosphate buffered saline (PBS), fetal bovine serum (FBS), L-glutamine, penicillin, streptomycin, trypsin (0.05% with ethylenediaminetetraacetic acid 0.53 mM), tetramethylrhodamine (TRITC)-labeled phalloidin, DRAQ5^TM^ were purchased from Life Technologies GmbH (Darmstadt, Germany). All other chemicals were purchased from Sigma-Aldrich Chemie GmbH (Munich, Germany), unless otherwise stated. All solutions were prepared using ultra-pure water supplied by a Milli-Q system (Millipore, Billerica, USA). HeLa cells (ATCC® CCL-2™) from the American Type Culture Collection (Manassas, Virginia, USA) were used in the experiments. All incubations were done at 37 °C, unless otherwise stated.

### Fabrication of anodic TiO_2_NT arrays

Before the anodization, titanium foils were degreased using a sonication process for 30 min in a mixed solution (acetone/ethanol/deionized water, at equal vol. ratio) and then dried using nitrogen stream. The anodization cell with an O-ring size of 6 cm was assembled to house a squared Ti foil (7 × 7 cm). The titanium foil was then fixed in the prepared anodization cell sealed by an O-ring and a back contact Cu-plate (area exposed to the electrolyte was 28.27 cm^2^). The assembled, two-electrode anodization cell with a platinum counter electrode (mesh type) placed 1.5 cm above the titanium surface was connected to a DC power source (PGU 200V-2A, IPS Elektroniklabor GmbH & Co. KG, Münster, Germany). The electrolyte composed of ammonium fluoride (0.27M) in glycerol/water (60/40 v/v) solution was added to the assembled anodization cell. Afterwards, a three-step anodization was employed that consisted of (i) 35V for 240 min, (ii) 5V for 10 min, and (iii) 35V for 60 min to generate weak points. The voltage and current were automatically changed (an ultrafast switching voltage), and then monitored and recorded in situ by means of EcmWin software (IPS Elektroniklabor GmbH & Co. KG).

### Ferric deposition into anodic TiO_2_NT arrays

As depicted in Fig. 1, a pure ferric solution (Ferrofluid-EFHI, Ferrotec Corporation, Bedford, USA, composed of 3–15% iron oxide (magnetite) and 6–30% oil-soluble dispersant in 55–91% distillates (petroleum), viscosity of 6 mPa·s, saturation magnetization 44 mT) was pipetted onto the surface of a TiO_2_NT array placed on a permanent magnet (NdFeB cylinder, Ø: 2 cm, length: 1 cm, 1.42T, FIXUM Creative Technology GmbH, Berlin, Germany). Note that the amount of ferric solution depends on the area of the array and should be adjusted in order to cover the surface of the array placed on the permanent magnet. After the internalization of the ferric fluid in the tubes, the organic stabilizer of the ferric solution was removed by performing annealing at 420 °C for 3 h.

### PEI-coated ferric/TiO_2_NT arrays

After the ferric deposition, the TiO_2_NT arrays were incubated with carbonyldiimidazole solution (100 mg/mL, chloroform) for 24 h at room temperature. Carbonyldiimidazole was used as a linker to covalently conjugate PEI polymer (200 kDa, 0.1% w/v ethanol) on the surface of carbonyldiimidazole activated TiO_2_NT arrays (incubated for 24 h at room temperature).

### Fabrication of FITC-conjugated magnetic TiO_2_NTs

FITC was conjugated on the surface of magnetic TiO_2_NTs (PEI-coated ferric deposited TiO_2_NTs). 10 μL of FITC solution (stock: 2 mg dissolved in 1 mL dimethyl sulfoxide = DMSO) was added to PEI modified magnetic TiO_2_NT arrays and incubated in the dark at room temperature. After the labeling reaction, the FITC-modified arrays were washed twice with water and ethanol, and then sonicated to obtain separate nanotubes.

### Fabrication of camptothecin loaded magnetic TiO_2_NTs

To load camptothecin into PEI/ferric/TiO_2_NT arrays for drug delivery system, the arrays were soaked overnight in camptothecin solution (5 mg/mL in dimethylformamide). The camptothecin-loaded arrays were then sonicated (~90 min) to obtain drug-loaded magnetic nanotubes. The nanocarriers were separated from the supernatant by centrifugation, rinsed (deionized water), and stored at 4 °C. The amount of absorbed camptothecin (Ex/Em wavelength: 340/440 nm) was measured with a fluorescence spectrophotometer (Fluorolog®-3, HORIBA Jobin Yvon Inc., Kyoto, Japan).

### Nanostructure characterization

SEM images were obtained with a high-resolution field-emission scanning electron microscope (FE-SEM, Hitachi S4800, Tokyo, Japan) by collecting backscattered electrons (10 kV beam energy under high vacuum 1 × 10^−5^ Pa). The ζ-potential (mV) of freestanding nanocarriers was obtained from the electrophoretic mobility measured by Laser Doppler Electrophoresis, using a Zetasizer Nano ZS90 instrument (Malvern Instruments, Malvern, UK) equipped with a 633 nm laser, using the Henry’s function.

X-Ray photoelectron spectroscopy (XPS, Physical Electronics 5600, Chanhassen, USA) was employed to study the surface chemistry of the nanocarriers at different stages (peaks were calibrated with C1s at 284.8 eV). X-ray diffraction was also used to measure the crystallographic structures of elongated anodic nanocarriers before and after the thermal treatment (420 °C for 3 h), using an X-ray diffractometer (XRD, Philips X’Pert, PANalytical B.V., Almelo, Netherlands) with Cu-kα radiation operated at 30 kV and 15 mA at a scan rate of 2^o^/min.

### Drug release

The camptothecin-loaded magnetic TiO_2_NTs were suspended at a concentration of 1.5 mg/mL in PBS (3 mL, pH 7.4) at 37 ± 0.5 °C in the dark. The supernatant (100 µL) was withdrawn at different release times (1, 2, 5, 8, 12, 24, and 36 h) and replaced with the same amount of fresh PBS medium. The amount of released camptothecin was then measured using a fluorescence spectrophotometer.

### Cell culture

HeLa cells (1 × 10^4^ per well) were seeded in a 96 well plate and incubated until confluency after 24 h. The cells were then treated with tubular TiO_2_ nanocarriers at different concentrations. To analyze the drug release under a magnetic force, two sets of samples (camptothecin-loaded and unloaded magnetic TiO_2_NTs) were dispersed in DMEM with 10% FBS, 100 U/mL penicillin, and 100 μg/mL streptomycin, and then added to the cells. The cells were incubated with each set of the nanocarriers for 30 min at different concentrations at 37 °C and 5% CO_2_ with and without employing a magnetic force. After incubation, the cells were rinsed twice with PBS (“washing step”) to remove all unbound nanocarriers followed by the replacement of fresh DMEM (without nanocarriers) in 5% CO_2_ at 37 °C for next 72 h. Afterwards, the viability of cells treated with nanocarriers and non-treated cells (control samples) was measured using the MTT assay^9^.

### Confocal microscopy

Cells were seeded at a density of 5 × 10^4^ cell/cm^2^ on glass coverslips, incubated for 24 h until confluency, and FITC-conjugated magnetic nanocarriers (200 µg/ml) were added. After incubation (see cell culture section), nanocarrier-treated and non-treated cells were fixed in a solution, containing 4% paraformaldehyde and DRAQ5^TM^ (Ex/Em wavelength: 646/697 nm) for 30 min according to the manufacturer’s protocol. Cells were then permeabilized with 0.05% Triton X-100 for 10 min followed by the addition of 10 μL (1 mg/mL in DMSO) TRITC-labeled phalloidin (Ex/Em wavelength: 540⁄565 nm) for staining the actin filaments in the cytoplasm. At the end, the treated cells were washed using PBS and mounted with fluorogel mounting reagent (ibidi GmbH, Martinsried, Germany). A confocal fluorescence microscope (Leica Microsystems, Wetzlar, Germany) was then employed to investigate the cellular binding of FITC-conjugated magnetic TiO_2_NTs.

### SEM of cells

Cells were grown on glass coverslips, nano-carrier-treated and fixed as described in the previous paragraph. The fixed cells were exposed to a gradual dehydration (a series of ethanol/water solutions (60% to 100% ethanol with 5% incremental increases). After drying, a sputter-coating process was employed to cover the cells with a thin layer of platinum (approximately 1 nm thickness). The cells were then imaged with SEM at 2 kV beam energy under high vacuum (1 × 10^−5^ Pa).

### Magnetic tweezer device

For targeted manipulation of single TiO_2_NTs, we used a magnetic tweezer device, consisting of needle-shaped iron core that can be magnetized using a solenoid. HeLa cells were seeded overnight in a 35 mm tissue culture dish and incubated with TiO_2_NTs for 30 min (see cell culture). Next, the cells were washed three times in culture medium to remove unbound nanotubes. The tweezer tip was placed approximately 10 µm in front of a membrane-bound tube, using a micromanipulator (Eppendorf InjectMan NI-2, Eppendorf AG, Hamburg, Germany). Tip and TiO_2_NTs position were monitored using a brightfield microscope (Leica Microsystems, Wetzlar, Germany). By applying solenoid current, magnetic flux is induced in the iron core material that ultimately results in a sharp gradient magnetic field that induces a magnetic dipole moment in TiO_2_NTs located close to the tweezer tip. Note that TiO_2_NTs were only selected for the measurement when they had a sufficient distance to other tubes in order to avoid interactions between the induced dipole moments. Before and after every measurement, the tweezer was degaussed by a sinusoidal decaying solenoid current to avoid hysteresis effects of the iron core. The maximum solenoid current, which could be applied before reaching field saturation, was 3A. To estimate the resulting magnetic force on TiO_2_NTs, we performed a calibration experiment with nanotubes mixed in silicone oil with known kinematic viscosity of 300 cSt. Using Stokes’ law, we estimated a magnetic force of 40 nN on TiO_2_NTs at a distance of 10 µm to the tweezer tip.

### Statistics

Statistical significance between populations was calculated by using one-way ANOVA followed by Tukey’s multiple comparison post-hoc analysis.

## Supplementary information

Supplementary Information

Supplementary Information
