# Supplementary material for: Anodic Titanium Dioxide Nanotubes for Magnetically Guided Therapeutic Delivery
Source: Sci Rep. 2019 Sep 17;9:13439. doi: 10.1038/s41598-019-49513-2 (PMC6748954; doi:10.1038/s41598-019-49513-2)
Supplement: Supplementary file 2 — Supplementary Information [file 41598_2019_49513_MOESM2_ESM.docx]

Supporting Information

Anodic Titanium Dioxide Nanotubes for Magnetically Guided Therapeutic Delivery

Morteza Hasanzadeh Kafshgari,^1,2,4^ Delf Kah,^1^ Anca Mazare,^2^ Nhat Truong Nguyen,^2^ Monica Distaso,^3^ Wolfgang Peukert,^3^ Wolfgang H. Goldmann,^1*^ Patrik Schmuki,^2*^ Ben Fabry^1*^

^1^Department of Physics, Biophysics Group, University of Erlangen-Nuremberg, 91052 Erlangen, Germany

^2^Department of Materials Science and Engineering, WW4-LKO, University of Erlangen-Nuremberg, Martensstrasse 7, 91058, Erlangen, Germany

^3^Institute of Particle Technology, University of Erlangen-Nuremberg, 91058 Erlangen, Germany

^4^Present address: Department of Engineering Physics, Polytechnique Montreál, Montreál, Quebec, H3C3A7, Canada

^*^Prof. Wolfgang H. Goldmann, PhD; Email: wgoldmann@biomed.uni-erlangen.de

^*^Prof. Dr. Patrik Schmuki; Email: schmuki@ww.uni-erlangen.de

^*^Prof. Dr. Ben Fabry; Email: bfabry@biomed.uni-erlangen.de

**Section S1: SEM micrographs of magnetic TiO_2_ tubular arrays**

| 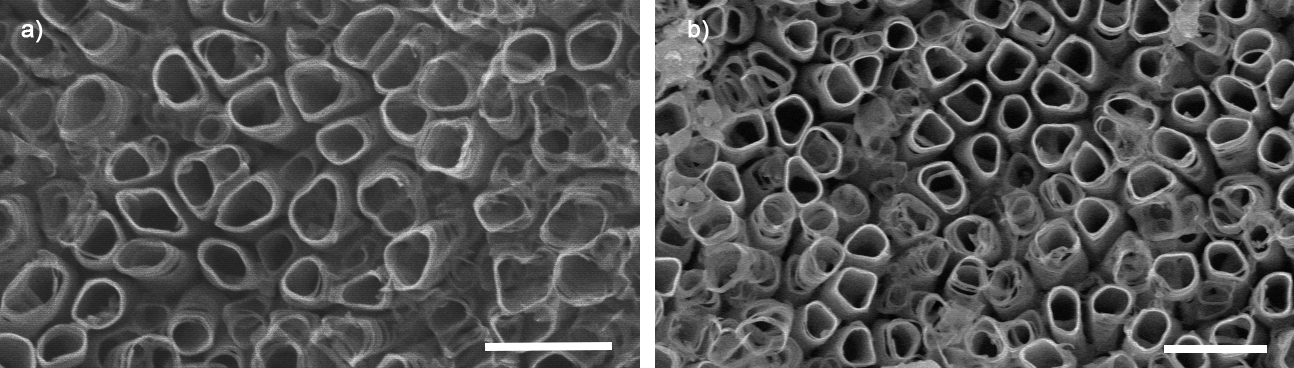 |
| --- |
| **Figure S1.** Representative SEM micrographs of the top view of (a) TiO_2_NTs and (b) Ferric/TiO_2_NTs. Scale bars show 500 nm. |

**Section S2: XPS characterization of magnetic TiO_2_NTs**

| 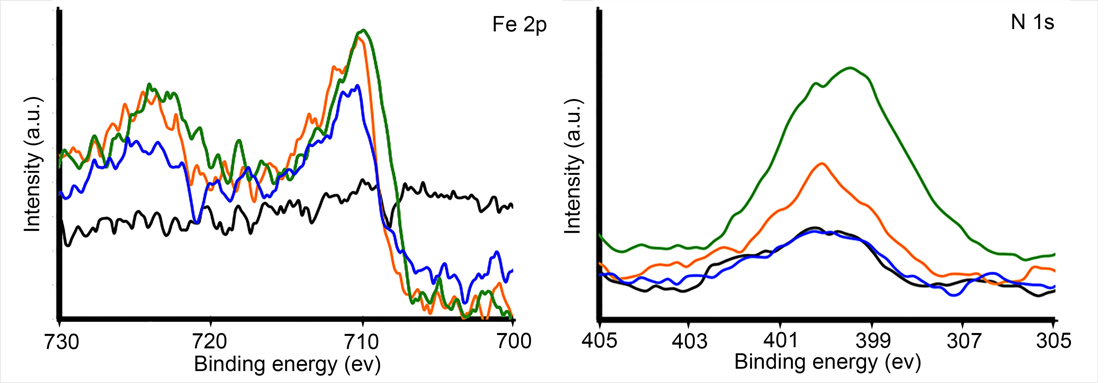 |
| --- |
| **Figure S2.** Overlay of XPS Fe 2p and N 1s spectra of TiO_2_NTs (black line), Ferric/TiO_2_NTs (blue line), carbonyldiimidazole modified ferric/TiO_2_NTs (orange line) and PEI-coated carbonyldiimidazole modified Ferric/TiO_2_NTs (magnetic TiO_2_NTs, green line). |

| 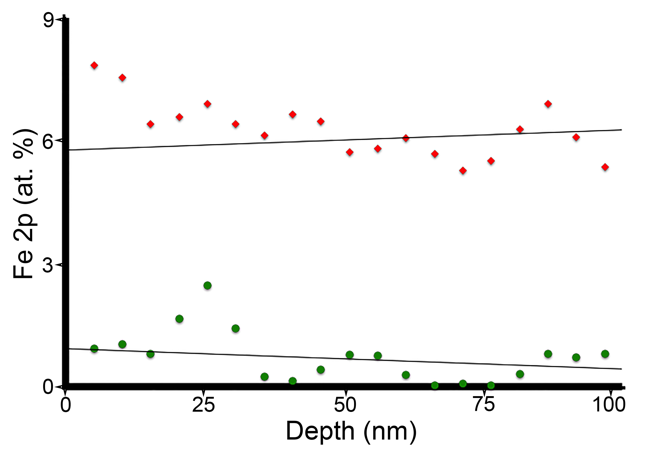 |
| --- |
| **Figure S3**. Depth profile of XPS Fe 2p spectra for the fabricated array of magnetic TiO_2_NTs (red dots) and unmodified TiO_2_NTs (green dots). |

**Section S3: An example of ridges connecting the anodic tubes**

| 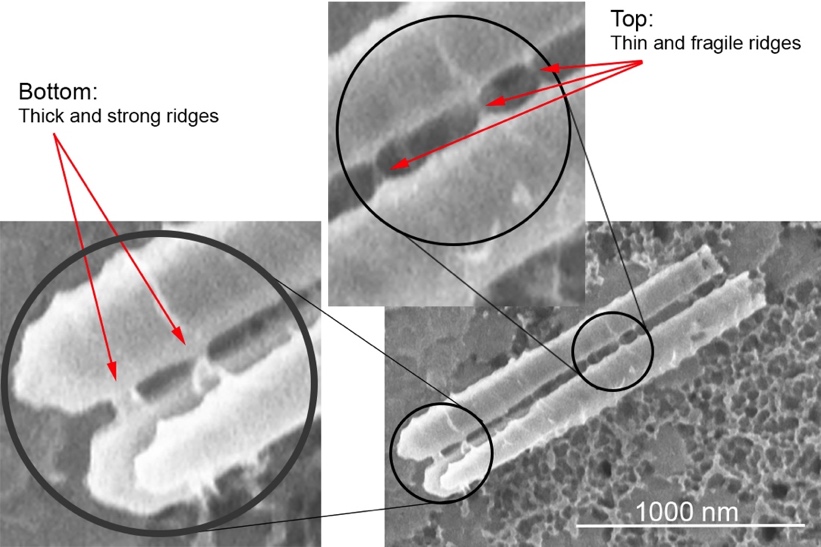 |
| --- |
| **Figure S4.** A representative SEM micrograph shows ridges of the double connected TiO_2_NTs. |

**Section S4: Cellular binding of magnetic TiO_2_NTs**

| 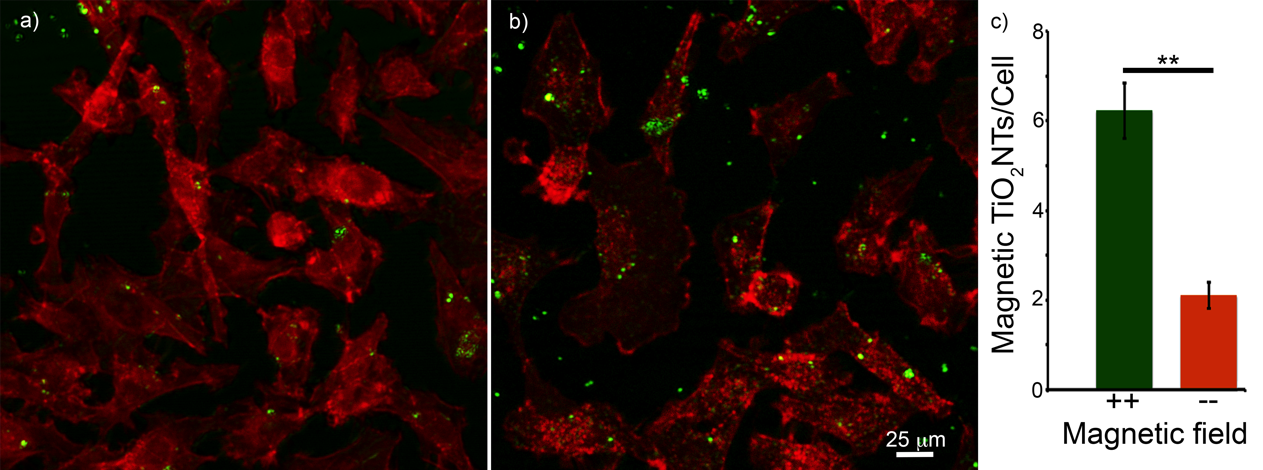 |
| --- |
| **Figure S5.** A representative fluorescence micrograph of the binding magnetic TiO_2_NTs into HeLa cells (a) without and (b) with using a magnetic field. FITC conjugated magnetic TiO_2_NTs appeared green, and the cell cytoskeleton was stained with phalloidin-TRITC (red). c) The average number of the bound magnetic TiO_2_NTs per HeLa cell with (green column, ++) and without (red column, --), using a magnetic force. Statistically significant cellular binding (one-way ANOVA followed by Tukey’s multiple comparison post hoc analysis) is marked (**P < 0.01).   \| 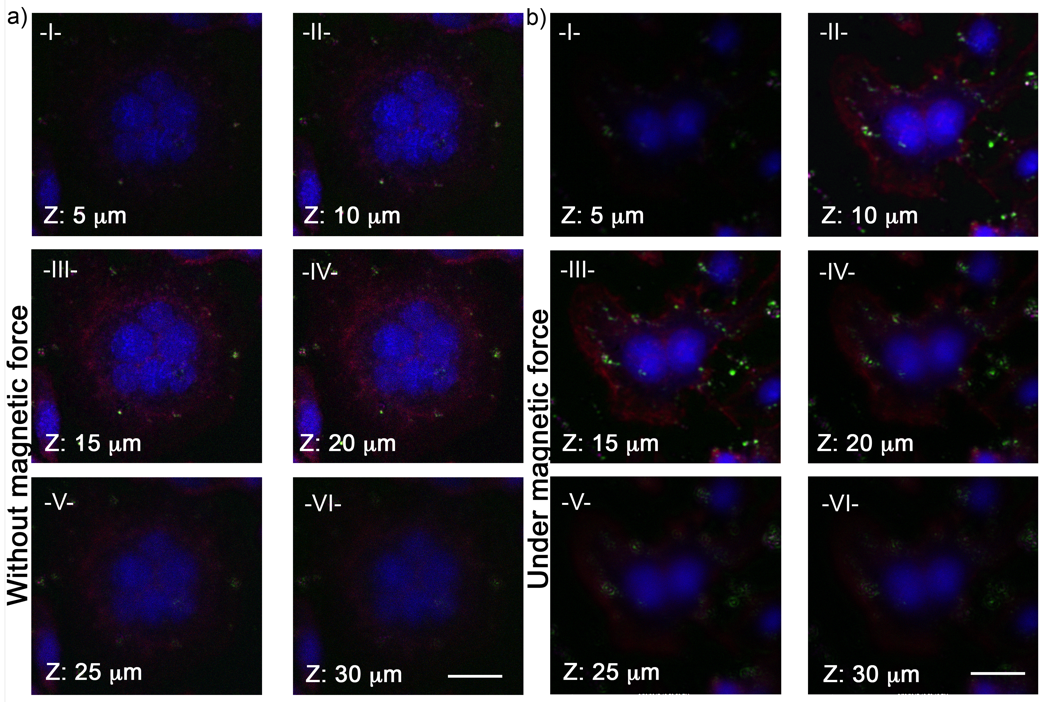 \| \| --- \| \| **Figure S6.** A representative fluorescence micrograph of the binding magnetic TiO_2_NTs into HeLa cells (a) without and (b) with using a magnetic field. FITC conjugated magnetic TiO_2_NTs appeared green, nuclei stained by means of DRAQ5^TM^ are shown blue, and the cell cytoskeleton was stained with phalloidin-TRITC (red). Roman numbers indicate different steps (5 µm) in the Z-direction. \| |

**Section S5: Movement of the bound magnetic TiO_2_NTs using a magnetic tweezer device**

*A supporting movie*

**Section S6: Cellular internalization of oligonucleotide-loaded magnetic TiO_2_NTs**

To load FAM-oligonucleotide (Fluorescein amidite labeled GAG GCT TTG ATC GTC AAG TTT (21mer)) into PEI/ferric/TiO_2_NT arrays (oligonucleotide loaded magnetic TiO_2_NTs) as a proof of concept for studying cellular attachment, the arrays were soaked into the oligonucleotide solution (5 ng/mL in ethanol) and incubated overnight (4 ^o^C). Following incubation, the oligonucleotide-loaded arrays were mildly sonicated for ~90 min to generate separated tubular nanocarriers (oligonucleotide-loaded magnetic TiO_2_NTs). The nanostructures were then collected under the static magnetic field, and supernatant was removed. The nanocarriers were washed (DI water) and separated from the supernatant by centrifugation. Oligonucleotide loaded magnetic TiO_2_NTs were collected by centrifugation and kept in the fridge at 4 ^o^C prior to experimentation. Prior to cellular uptake, oligonucleotide loaded magnetic TiO_2_NTs were prepared by a mild sonicating in the DMEM in the dark. Afterwards, the magnetic TiO_2_NTs were used to treat HeLa cells according to the sections “cell culture and confocal microscopy”.

| 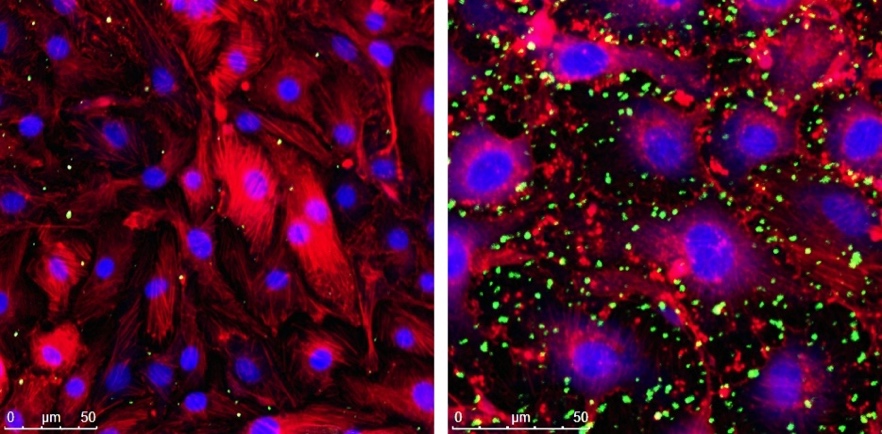 |
| --- |
| **Figure S7.** Cellular binding of FAM-oligonucleotide magnetic TiO_2_NTs by HeLa cells 30 min after first NP exposure: (a) Control HeLa cells without and (b) with using a magnetic force. The cell nucleus was stained with DRAQ5^TM^ (shown blue), FAM-oligonucleotide magnetic TiO_2_NTs appeared green, and the cell cytoskeleton was stained with phalloidin-TRITC (red) (representative data). |

| 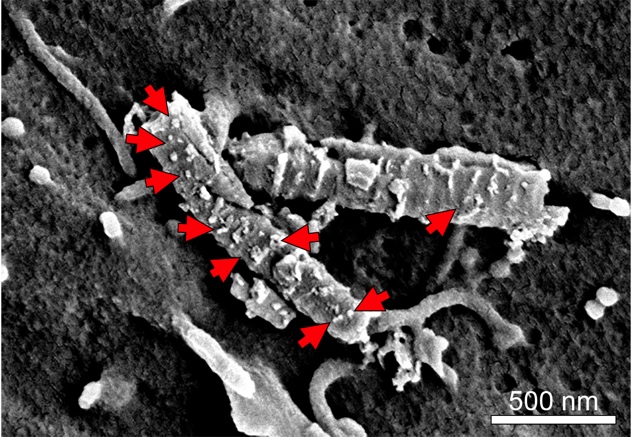 |
| --- |
| **Figure S8.** A representative SEM micrograph of the internalizing magnetic TiO_2_NTs into HeLa cells. Arrows show iron nanoparticles on the surface of the magnetic TiO_2_NTs. |
